# Insight into the Mechanism for the Emergence of Thermally Stable Reflection Colors from Cholesteric Liquid Crystals of Etherified Ethyl Cellulose Derivatives and Methacrylic Acid

**DOI:** 10.3390/molecules30132839

**Published:** 2025-07-02

**Authors:** Wakako Kishi, Naoto Iwata, Seiichi Furumi

**Affiliations:** Department of Chemistry, Graduate School of Science, Tokyo University of Science, 1-3 Kagurazaka, Shinjuku, Tokyo 162-8601, Japan; 1325539@ed.tus.ac.jp

**Keywords:** ethyl cellulose, cholesteric liquid crystal, Bragg reflection, small-angle X-ray scattering, phase separation

## Abstract

Ethyl cellulose (EC) and its derivatives are known to exhibit the cholesteric liquid crystal (CLC) phase with visible light reflection in a lyotropic manner after adding appropriate solvents. Generally, the reflection peak of conventional CLCs is easily wavelength shifted by temperature. However, our previous study showed that the reflection wavelength can be maintained even after heating for the lyotropic CLCs of completely pentyl-etherified EC derivatives with methacrylic acid (MAA). However, the emergence of thermally stable reflection colors still remains obscure in the mechanism at the mesoscopic scale. In this study, we evaluated the temperature dependence of the reflection wavelength for the lyotropic CLCs of a series of completely etherified EC derivatives possessing different alkoxy chains by addition of MAA. It was found that butyl- or pentyl-etherified EC derivatives are suitable for preparation of the lyotropic CLCs with visible Bragg reflection, whereas visible light reflection cannot be observed for the other mixtures of propyl- and hexyl-etherified EC derivatives with MAA. Furthermore, it turned out that lyotropic CLCs of pentyl-etherified EC derivatives with MAA show the smallest temperature dependence of their reflection wavelength. Based on the results of ultra-small-angle X-ray scattering (USAXS) and small-angle X-ray scattering (SAXS) measurements of CLC films, we presumed that the emergence of thermally stable reflection colors from the lyotropic CLCs of pentyl-etherified EC derivatives with MAA arises from their phase separation at the mesoscopic scale by changing the temperature.

## 1. Introduction

The cholesteric liquid crystal (CLC) phase is generally composed of nematic liquid crystal layers of calamitic liquid crystal molecules with intrinsic molecular chirality, that is, optical activity; however, their orientational directors continuously rotate in a periodic manner at the macroscopic scale so as to self-organize the helical molecular assemblages with left- or right-handed sense by the helical twisting power of chiral molecules [1]. These molecular assemblages give rise to the periodic modulation in refractive index in the CLC medium, thereby leading to the emergence of light reflection at a specific wavelength through the light interference. This light reflection phenomenon by CLCs can be regarded as a kind of Bragg reflection. The reflection peak wavelength (*λ*) is numerically expressed as the following equation:

*λ* = *n*·*p* (1)

where *n* and *p* mean the average refractive index and the helical pitch length of CLC, respectively [2]. When the helical pitch length of the CLC medium is equally matched to several hundred nanometers, the light reflection phenomenon can be visualized as the reflection colors of blue, green and red. Due to the liquid crystallinity, the helical pitch length of CLC exhibits sensitivity to variations in temperature [3], electric field [4], magnetic field [5], mechanical stress [6], and photo-irradiation [7]. Such tunability of the CLC helical pitch length by the external stimuli allows for the on-demand control of reflection peak wavelength, enabling the technological applications to reflective color displays [8], full-color recording media [9], and so forth. Furthermore, the research on mirrorless lasing from CLCs has also attracted considerable interest from the perspective of photonic crystals [10,11,12,13].

However, as mentioned above, the CLC helical pitch length is very vulnerable to subtle temperature fluctuation due to the fluidity of liquid crystallinity. Eventually, the reflection peak wavelength is readily shifted in wide wavelength ranges by changing slight temperature [14,15,16]. In order to keep the reflection peak wavelength, specific temperature-controller is needed in the experiment. Moreover, conventional CLC materials are predominantly synthesized from finite petroleum resources, raising the natural resource issues for the future generation.

To address these emergency issues, we paid attention to cellulose derivatives. Cellulose and its derivatives have gained considerable interest as biomass resources, arising from the safety, biocompatibility, and abundance. This is because cellulose is a major component of plants. In addition, another unique property of cellulose and its derivatives is the appearance of CLC phase by dissolving or suspending them in appropriate solvents [17,18]. The mesophase is dependent on the concentration of cellulose derivatives, which is called the lyotropic liquid crystal phase. Taking account of the outstanding features, numerous studies have been conducted aiming for their application to functional materials of cellulose derivatives that can contribute to the realization of a sustainable society.

**Figure 1:**
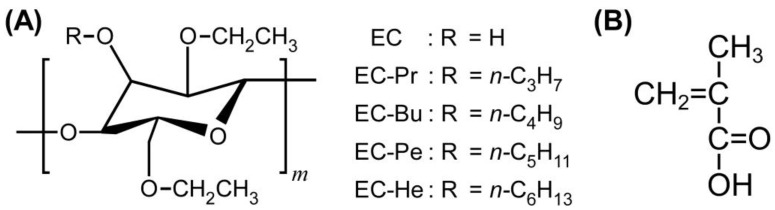
(**A**) Chemical structures of ethyl cellulose (EC) and its derivatives. (**B**) Chemical structure of methacrylic acid (MAA).

Ethyl cellulose (EC), as depicted in Figure 1A, is one of representative cellulose derivatives. In general, EC is prepared by reacting natural cellulose with ethylene oxide. Due to its safety, EC is nowadays available in not only the medical realm such as pharmaceutical agents [19], but also industrial realm such as components in optoelectronic devices [20]. More interestingly, EC has been reported as biodegradable materials for the contribution toward the circular economy [21].

EC is known to exhibit the lyotropic CLC phase with visible light reflection [22,23,24,25]. For instance, the solutions of pristine EC in organic solvents such as chloroform [23,24] or acrylic acid [26] exhibit the lyotropic CLC phase. As another precedent, esterified EC derivatives exhibit the lyotropic CLC phase when dissolved in solvents at appropriate concentrations [23,24,25]. The mechanism of the Bragg reflection color change in CLCs from EC or its derivatives depending on their concentration can be explained by the difference in the helical pitch length of lyotropic CLC, corresponding to *p* in Equation (1). Therefore, the reflection peak wavelength of lyotropic CLCs is significantly dependent on the concentrations of EC or its derivatives [26]. In our previous report, we succeeded that the reflection peak wavelength of lyotropic CLCs of completely pentyl-etherified EC derivatives with methacrylic acid (MAA; Figure 1B) can be maintained irrespective of temperature in the range between 30 °C and 110 °C [27]. This phenomenon is quite interesting. This is because CLCs generally exhibit the temperature-induced shift of reflection peak in the wide wavelength range as the thermotropic behavior. Therefore, the mechanism for the emergence of thermally stable Bragg reflection colors from the lyotropic CLCs of EC derivatives and MAA still remains unclear at the mesoscopic scale. In addition, the temperature dependences of lyotropic CLCs from EC derivatives substituted with other alkoxy groups have not been reported yet even though it may play a crucial role to the reflection peak wavelength of CLCs similar to the case of etherified derivatives of hydroxypropyl cellulose [28].

In this study, we evaluated the temperature dependence of reflection peak wavelength of lyotropic CLCs from the etherified EC derivatives and MAA. As presented in Figure 1A, a series of etherified EC derivatives possessing propyloxy (EC-Pr), butyloxy (EC-Bu), pentyloxy (EC-Pe), or hexyloxy groups (EC-He) were prepared in order to unravel the effect of their alkoxy side chain length of etherified EC derivatives on the light reflection properties. Although visible light reflection could not be observed for the mixtures of EC-Pr and EC-He with MAA, it turned out that EC-Bu and EC-Pe are suitable for the preparation of lyotropic CLCs with visible Bragg reflection. Furthermore, lyotropic CLCs of EC-Bu with MAA exhibited the relatively independence of reflection peak wavelength upon the changing the temperature, as compared to those from EC-Pe and MAA, suggesting that EC-Pe is a prime candidate to prepare the lyotropic CLC with a thermally stable reflection color. Ultra-small-angle X-ray scattering (USAXS) and small-angle X-ray scattering (SAXS) measurements of the cured CLC films revealed that the appearance of spherical scatterers by elevating the temperature, arising from the thermally induced phase separation between EC derivatives and MAA at the mesoscopic scale. Based on these experimental results, we presumed that the emergence of thermally stable reflection colors from lyotropic CLCs of EC-Pe with MAA can be ascribed to their constant CLC helical pitch length induced by the phase separation at the mesoscopic scale by heating treatment.

## 2. Results and Discussion

### 2.1. Characterization of EC Derivatives

Four types of completely etherified EC derivatives such as EC-Pr, EC-Bu, EC-Pe, and EC-He were prepared by the Williamson ether synthesis according to our previously reported procedure in order to elucidate the side chain effect of etherified EC derivatives on the light reflection properties of lyotropic CLCs with MAA (Figure 1A) [27]. All of the EC derivatives were obtained as white powder, and did not exhibit thermotropic liquid crystallinity on their own unlike etherified hydroxypropyl cellulose derivatives [28].

The etherified EC derivatives were characterized by measuring both Fourier transform infrared (FT-IR) and nuclear magnetic resonance (NMR) spectra to confirm that hydroxy groups of pristine EC are completely substituted with alkoxy groups. Figure S1A in the Supplementary Materials shows the comparison of FT-IR spectra between pristine EC and etherified EC derivatives. As compared these spectra, the pristine EC showed an intense peak from the O-H stretching vibration of the hydroxy groups in a broad wavenumber range from 3000 cm^−1^ to 3600 cm^−1^. In contrast, the intense peaks around 3000–3600 cm^−1^ were not observed for the completely etherified EC derivatives so as to be broad bands with weak intensities. The experimental results imply that the hydroxy groups of pristine EC are completely etherified regardless of the difference in the chain length of alkoxy groups. Figure S1B in the Supplementary Materials represents the ^1^H-NMR spectra of etherified EC derivatives in CDCl_3_. From the ^1^H-NMR spectra, the *DS* values, that is, the degree of substitution with alkoxy groups, are quantitatively estimated according to the following equation [27]:

(2) $$DS=\frac{A\left(7+5MS\right)}{3W-\left(2x+1\right)A}$$

where *A* is the integrated value of the signal peak “*a*” assigned to the protons of the terminal methyl group, *W* is the sum of the integrated values of all protons in EC derivatives, and *x* is the number of carbon atoms in alkoxy groups. For instance, *x* corresponds to 3 in the case of EC-Pr. As will be mentioned in Section 3.1, we adopted the *MS* value of 2.50 from the ^1^H-NMR spectrum of pristine EC. By applying the experimental results to Equation (2), the *DS* values of all etherified EC derivatives were estimated to be 0.50, which also assured that the hydroxy groups of pristine EC are completely etherified for EC-Pr, EC-Bu, EC-Pe, and EC-He.

### 2.2. Reflection Properties of Lyotropic CLCs of EC Derivatives with MAA

When the lyotropic CLCs of EC-Pr, EC-Bu, EC-Pe, and EC-He with MAA were prepared, the light reflection properties were found to be different behavior as the alkoxy side chains. Although we found that EC-Bu and EC-Pe are suitable for the preparation of lyotropic CLCs with visible Bragg reflection, the reflection color was not observed for the lyotropic CLCs of EC-Pr with MAA at the concentration of 35–60 wt%. As measuring the transmission spectra of the lyotropic CLCs, no peak was detected in the visible wavelength region of 400–800 nm, which was the detectable wavelength range of the spectroscopy used in this study. As observed using polarized optical microscope (POM) under cross-Nicols, the optical anisotropy appeared in the lyotropic CLCs of EC-Pr with MAA. It is plausible that the lyotropic CLC of EC-Pr with MAA might reflect ultraviolet light or infrared light. Similarly, the lyotropic CLCs of EC-He with MAA at the concentration of 60–75 wt% showed no reflection color even though they showed optical anisotropy by the POM observation. From the previous report on the thermotropic CLCs of etherified derivatives of hydroxypropyl cellulose, the reflection peak wavelength red-shifts with the increase in the chain length of alkoxy groups substituted to the hydroxy groups of hydroxypropyl cellulose [28]. Therefore, it can be assumed that the lyotropic CLCs of EC-Pr and EC-He dissolved in MAA reflect ultraviolet and infrared light, respectively.

**Figure 2:**
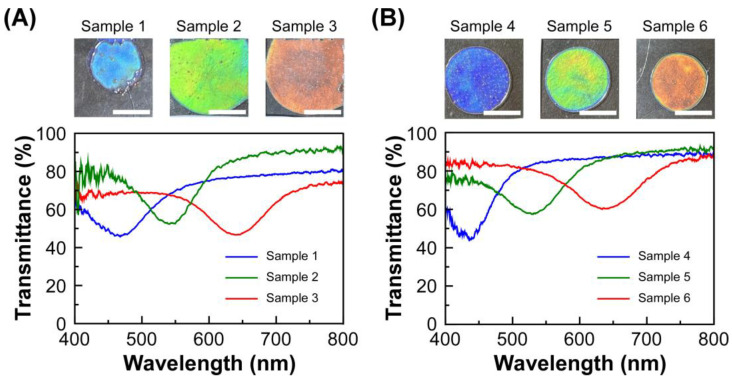
Changes in transmission spectra of cells of lyotropic CLCs of EC-Bu or EC-Pe with MAA as a function of the concentration of EC derivatives. The measurement temperature was set at 30 °C. Insets show the reflection images of CLC cells, and the white scale bars denote 5.0 mm. (**A**) EC-Bu in MAA, Samples 1–3; (**B**) EC-Pe in MAA, Samples 4–6.

To investigate the temperature dependence of light reflection properties, six types of lyotropic CLCs were prepared from EC-Bu or EC-Pe. The preparation conditions and sample definition of lyotropic CLCs from EC-Bu or EC-Pe are summarized in Table 1. Figure 2 shows the transmission spectra of lyotropic CLCs of EC-Bu or EC-Pe, which were measured at 30 °C. When EC-Bu was completely dissolved in MAA at the concentration range of 59.2–61.5 wt%, the mixtures of EC-Bu and MAA showed reflection colors as blue, green, and red by the formation of the lyotropic CLC phase (Figure 2A). The reflection wavelength continuously shifted to the longer wavelength side as diluting the concentration of EC-Bu in MAA, arising from the increase in the helical pitch length of CLC. Similar light reflection properties were observed for the lyotropic CLCs of EC-Pe with MAA at 67.4–69.4 wt% (Figure 2B). The reflection color also changed to blue, green, and red in the descending order of the concentration of EC-Pe by the red-shift of reflection peak wavelength. As described in the Introduction part, the reflection wavelength of lyotropic CLCs of EC-Pe with MAA are unlikely to be changed upon the heating process. Since the detectable range of our transmission spectral measurement system is 400–800 nm, it is essential to prepare the lyotropic CLCs with visible Bragg reflection for the fair comparison of their reflection wavelength upon the heating process. From this reason, we focused on the lyotropic CLCs of EC-Bu or EC-Pe with MAA to explore the temperature-dependence of their reflection peak wavelength.

### 2.3. Temperature Dependence of the Reflection Properties of the Lyotropic CLCs from EC Derivatives and MAA

**Figure 3:**
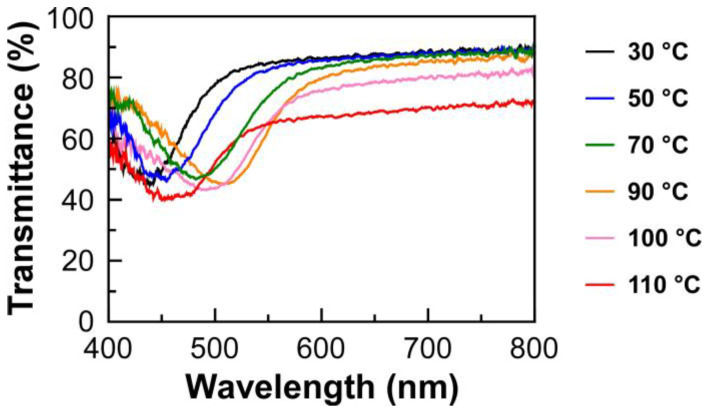
Changes in transmission spectrum of Sample 4 upon the heating process from 30 °C to 110 °C. The transmission spectral measurements were conducted at the temperature intervals of 10 °C. In this figure, only the transmission spectra at 30, 50, 70, 90, 100, and 110 °C are shown for reader’s clarity.

The transmission spectrum measurements were conducted for the lyotropic CLCs of EC-Bu or EC-Pe with MAA upon heating the process from 30 °C to 110 °C. The isotropic phase transition temperature was found to be ~150 °C for all samples. Furthermore, the result suggested that the lyotropic CLCs undergo no structural deterioration upon heating, as evidenced by the negligible change in their transmission spectra after the measurements. As an example, Figure 3 shows the changes in transmission spectrum of Sample 4, which corresponds to the lyotropic CLC of EC-Pe in MAA at the EC-Pe concentration of 69.4 wt%, as a function of the temperature upon the heating process. The reflection peak wavelength red shifted upon heating from 30 °C up to 90 °C. However, interestingly, the reflection peak wavelength blue shifted as elevating the temperature over 90 °C. At this time, the baseline of the transmission spectrum became lower than those measured below 90 °C. For instance, the light transmittance at longer wavelength of 800 nm drastically decreased from ~90% to ~65% by rising the temperature from 90 °C to 110 °C. Although it is obvious that the helical molecular assemblage of lyotropic CLC is maintained because the reflection peak can be clearly observed even in this temperature range, the decrease in light transmittance upon elevating the temperature strongly suggested the structural change in the CLC helical molecular assemblage at the mesoscopic scale. Such tendencies were also observed for transmission spectra of the other lyotropic CLCs upon the heating process (Supplementary Materials, Figure S2). The plausible mechanism for the changes in the CLC helical molecular assemblage will be discussed in the proceeding section.

**Figure 4:**
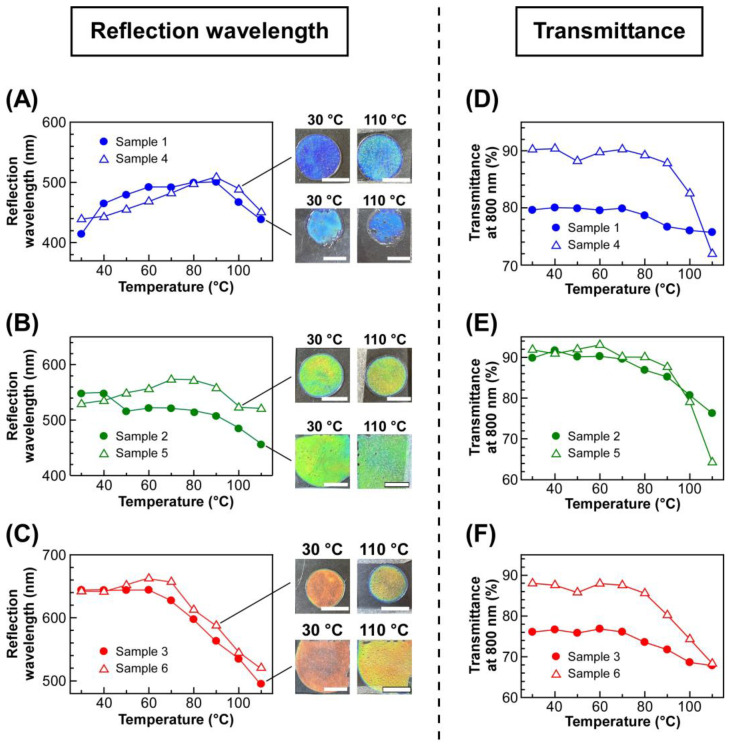
(Left panels) Changes in reflection peak wavelengths of lyotropic CLCs of EC-Bu or EC-Pe with MAA upon heating. (**A**) Lyotropic CLCs exhibiting a red reflection color, Samples 1 and 4. (**B**) Lyotropic CLCs exhibiting a green reflection color, Samples 2 and 5. (**C**) Lyotropic CLCs exhibiting a red reflection color, Samples 3 and 6. Insets represent the reflection images of CLCs, and the white scale bars are 5 mm. (Right panels) The change in light transmittances at 800 nm of the lyotropic CLC of EC- Bu or EC-Pe with MAA. (**D**) Lyotropic CLCs exhibiting a red reflection color, Samples 1 and 4. (**E**) Lyotropic CLCs exhibiting a green reflection color, Samples 2 and 5. (**F**) Lyotropic CLCs exhibiting a red reflection color, Samples 3 and 6.

To quantitatively analyze the changes in transmission spectra of lyotropic CLCs by changing the temperature, the reflection wavelength and light transmittance at 800 nm of each CLC were plotted against the temperature (Figure 4). The left panels of Figure 4 show the plots of the reflection wavelength of lyotropic CLCs of EC-Bu or EC-Pe exhibiting a blue reflection color (Figure 4A), green (Figure 4B), and red (Figure 4C), upon heating from 30 °C in a stepwise manner. As shown in Figure 4A, although the reflection wavelength of Sample 1 and Sample 4 continuously shifted to the longer wavelength side, it began to shift to the shorter wavelength side with increasing the temperature from 90 °C to 110 °C. However, it should be noted that the reflection color can be recognized as blue by naked eyes because of the relatively small wavelength shift of reflection peak. Therefore, it was indicated that EC derivatives that can make lyotropic CLCs with thermally stable reflection colors is not only limited to EC-Pe but also to EC-Bu. In fact, the reflection wavelength of Sample 2 and Sample 5 was maintained especially in the temperature range of 30–90 °C. Although the reflection wavelength slightly blue shifted when heated above 90 °C, the reflection color change is hardly recognized by naked eyes (Figure 4B). In the same manner, Sample 3 and Sample 6 showed exactly same tendency except that the blue-shift of reflection wavelength began at 70 °C, which was much lower than Sample 2 and Sample 5 (Figure 4C). Among the lyotropic CLCs exhibiting blue and green colors, Sample 3 and Sample 6 displayed a relatively large shift of reflection peak wavelength. This can be ascribed to the increase in the mobility of CLC arising from the decrease in the concentration of etherified EC derivatives when preparing lyotropic CLCs. To quantitatively discuss the shift of reflection wavelength of the lyotropic CLCs, the following equation was adopted to calculate the average wavelength shift of the reflection peak at the temperature range of 10 °C (*λ*_shift_/10 °C).

*λ*_shift_/10 °C = {(*λ*_110°C_ − *λ*_30°C_)/(110 °C − 30 °C)} × 10 °C (3)

where *λ*_30°C_ and *λ*_110°C_ are the reflection wavelength of lyotropic CLC at 30 °C and 110 °C, respectively [27]. The *λ*_shift_/10 °C values are summarized in the fifth column of Table 1. Because the λ_shift_/10 °C values for lyotropic CLCs of EC-Bu with MAA (Samples 1–3) were larger than those of EC-Pe with MAA (Samples 4–6), it was indicated that EC-Pe is much more adequate to prepare lyotropic CLC with a thermally stable reflection color.

As mentioned in the Section 2.2, the light transmittance of lyotropic CLCs decreased at elevated temperature. The right panels of Figure 4 show the changes in light transmittances at 800 nm for the lyotropic CLCs of EC-Bu or EC-Pe with MAA, exhibiting a blue reflection color (Figure 4D), green (Figure 4E), and red (Figure 4F), upon heating from 30 °C. The light transmittance of Sample 4 drastically decreased from ~90% to ~70% when the temperature exceeded 90 °C (Figure 4D, closed circles). Notably, this temperature agreed well with the temperature at which the reflection wavelength became maximum (Figure 4A, closed circles). Although the decrease in light transmittance was not clearly observed for Sample 1, the light transmittance at 90 °C was lower than that at 70 °C. These results indicated the temperature range can be divided into two regions; one is 30–90 °C, where the red-shift of reflection wavelength is observed, and the transmittance is maintained. The other is 90–110 °C, where the reflection wavelength blue-shifts and a decrease in light transmittance is observed. Similarly, the light transmittance of Samples 2 and 5 decreased above 90 °C, where the reflection wavelength began to blue-shift (Figure 4E). Furthermore, Sample 3 and Sample 6 also exhibited a similar tendency except that the infection points of the plots shifted to 70 °C (Figure 4F). These results strongly implied that internal structure of the lyotropic CLCs of etherified EC derivatives with MAA drastically changes throughout the temperature range of 30–110 °C. Therefore, the thermally stable reflection color of the lyotropic CLCs could not be ascribed to the stability of the internal structure upon heating.

### 2.4. USAXS and SAXS Measurements of UV-Cured Film from Lyotropic CLCs

To investigate the mechanism for the emergence of thermally stable Bragg reflection colors of CLCs from etherified EC derivatives and MAA, we conducted USAXS and SAXS measurements using the synchrotron radiation. This is because SAXS measurements have been widely used for the mesoscopic structure analysis of polymer-dispersed liquid crystals [29], and phase separation behavior of polymer solutions [30,31]. Based on these precedents, we presumed that the difference in scattering length density between EC derivatives and MAA is large enough to obtain clear SAXS patterns.

However, when conducting SAXS measurements, another concern about the evaporation of MAA from the lyotropic CLC was raised. In general, the reflection peak wavelength of lyotropic CLCs are drastically affected by the evaporation of solvent, resulting in difference in the internal structure of CLCs. To solve this problem, we conducted USAXS and SAXS measurements at room temperature for CLC films prepared by irradiation with ultraviolet (UV) light at 365 nm to the lyotropic CLCs of EC derivatives with MAA at 30 °C, 70 °C, or 110 °C. These solid-state CLC films might maintain their internal helical molecular assemblages of lyotropic CLCs at each curing temperature because of the progress of photopolymerization of MAA and subsequent cross-linking via hydrogen bonds. This is also evident from the fact that the reflection peak wavelengths of lyotropic CLCs and the films prepared by UV-irradiation are almost identical.

**Figure 5:**
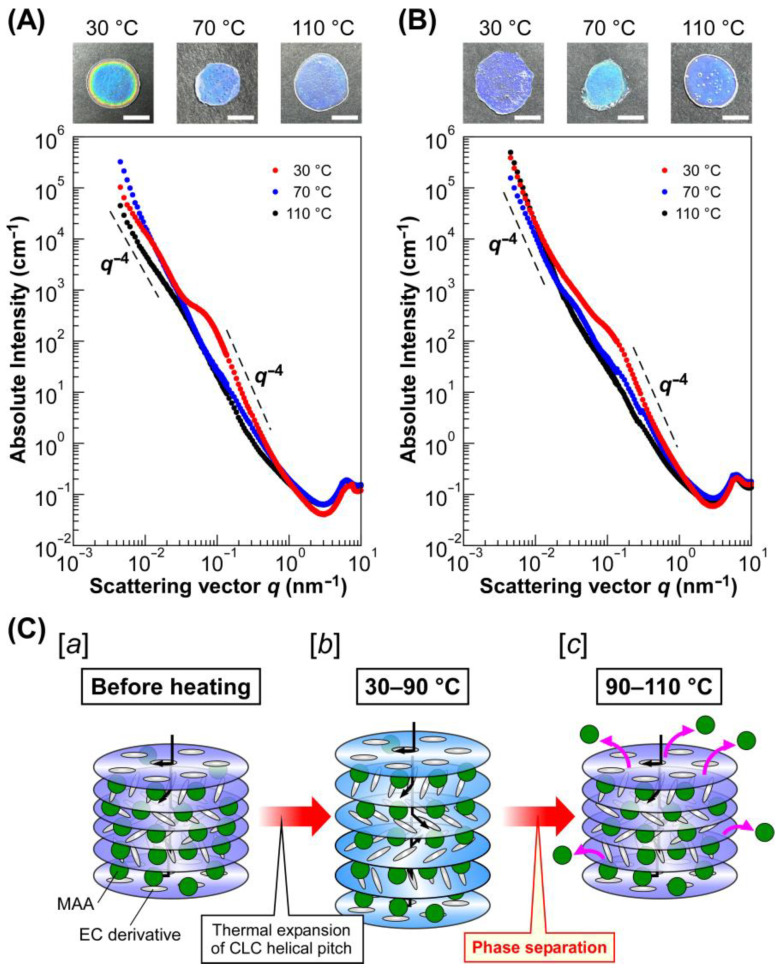
USAXS-SAXS intensity profiles of UV-cured films exhibiting blue reflection colors and plausible mechanism for the emergence of thermally stable Bragg reflection colors from lyotropic CLC of EC derivatives with MAA. (**A**,**B**) USAXS-SAXS intensity profiles of the solid-state CLC films from Sample 1 (**A**) and Sample 4 (**B**), which were cured with UV light at 30 °C (black circles), 70 °C (blue circles), and 110 °C (red circles). The insets represent the reflection images of UV-cured CLC films, and the white scale bars denote 5 mm. (**C**) Schematic illustration of structural changes in CLC from EC derivatives and MAA upon the heating process. [***a***] CLC structure before heating; [***b***] the CLC helical molecular assemblage at 30–90 °C, where reflection wavelength slightly red-shifts by thermal expansion of CLC helical pitch; [***c***] the CLC helical molecular assemblage at 90–110 °C, where MAA molecules expelled from the CLC helical molecular assemblage so as to induce the thermally induced phase separation at the mesoscopic scale.

Figure 5 shows the intensity profiles of UV-cured CLC films exhibiting a blue refection color, which were prepared by using EC-Bu (Figure 5A) and EC-Pe (Figure 5B). These profiles were obtained by calculating the absolute intensity by using the standard of glassy carbon and plotting against the scattering vector (*q*). A shoulder-like peak was observed at *q* = ~0.03 nm^−1^ in the profiles of the CLC film from Sample 1 cured at 30 °C (Figure 5A, black circles). A similar shoulder-like peak was also observed at almost the same *q* value for the intensity profiles of the film from Sample 1 cured at 70 °C (Figure 5A, blue circles). Interestingly, this peak became apparent and shifted to *q* = ~0.10 nm^−1^ in the profile for the CLC film cured a 110 °C (Figure 5A, red circles). Furthermore, the intensity became proportional to *q*^−4^ in the *q* range of 0.10–0.50 nm^−1^ and below 0.02 nm^−1^, suggesting the appearance of spherical scatterers. The size of spherical scatterers (*d*) can be approximated by the following equation:

(4) $$d=\frac{2\pi }{q_{s}}$$

where *q_s_* is the scattering vector at the shoulder-like peak. Therefore, it can be assumed that spherical scatterers with diameters of ~120 nm are formed in Sample 1 as cured at 110 °C. It should be noted that these scatterers are aggregated because the Guinier region was not clearly observed and the intensity continuously increased with a decrease in *q*, suggesting the existence of extremely large scatterers that cannot detected by the USAXS measurements, similar to the intensity profiles of composite materials consisting of polymeric matrices with aggregated inorganic fillers [32]. Such changes in USAXS-SAXS intensity profiles were also observed for the CLC films from EC-Pe. As shown in Figure 5B, the intensity profiles of the CLC films of Sample 4 cured at 30 °C and 70 °C. However, a shoulder like peak appeared at *q* = ~0.15 nm^−1^ for the CLC film cured at 110 °C. Therefore, it was found that the scatterers with a size of ~80 nm exist in the CLC film. Furthermore, the emergence of a shoulder-like peak in USAXS-SAXS intensity profiles upon increasing the temperature were also observed for the CLC films fabricated from Samples 2, 3, 5, and 6 (Supplementary Materials, Figure S3). Although the USAXS-SAXS intensity profiles were differed by curing temperatures and the reflection wavelength of the films due to changes in scattering length density difference or in the volume fraction of the scatterer, each profile had two regions where the intensity became proportional to *q*^−4^. Therefore, it was indicated that the formation of aggregated spherical scatters upon increasing the temperature is irrelevant to the chemical structure of EC derivatives or the concentration of lyotropic CLCs with MAA. By considering the fact that X-ray is scattered by differences in electron density of the samples, we presumed that the internal helical molecular structure of lyotropic CLCs of EC derivatives with MAA is greatly transformed by changing the temperature.

This hypothesis can be also supported by the POM observation of lyotropic CLCs (Supplementary Materials, Figure S4). As observing the POM images of lyotropic CLCs of EC-Bu or EC-PE with MAA exhibiting blue reflection colors at 30 °C, 70 °C, and 110 °C, all of the POM images were bright under cross-Nicols. Therefore, the lyotropic CLCs showed optical anisotropy arising from the liquid crystallinity [26]. However, the POM images of EC-Bu with MAA taken at 110 °C (Supplementary Materials, Figure S4C) and EC-Pe with MAA at 110 °C (Supplementary Materials, Figure S4F) showed a different morphology with spherical domains, suggesting the drastic changes in CLC helical molecular assemblages [33]. The size of spherical domains was estimated to be ~20 µm for the lyotropic CLC of EC-Bu with MAA, and ~30 µm for that of EC-Pe with MAA. Therefore, the lack of Guinier region in the USAXS-SAXS intensity profiles is reasonable because the size of scatterers, that is, domains observed by the POM, is extremely large to be measured by the USAXS.

### 2.5. Plausible Mechanism for the Emergence of Thermally Stable Bragg Reflection Colors from Lyotropic CLC of EC Derivatives and MAA

Based on the results mentioned in Section 2.3 and Section 2.4, we propose herein a mechanism for the emergence of thermally stable Bragg reflection colors from lyotropic CLCs of completely etherified EC derivatives with MAA. After heating the lyotropic CLCs of completely etherified EC derivatives with MAA, the CLC helical pitch length tends to expand by the thermodynamically induced increase in the mobility of CLC media (Figure 5C, image *b*). This is evident from the experimental fact that the red-shift of the reflection peak wavelength was observed from 30 °C to ~90 °C (Samples 1, 2, 4, and 5) or ~70 °C (Samples 3 and 6). At this stage, the absence of the change in the USAXS-SAXS profiles at 30 °C and 70 °C suggests that no significant structural change happens in the lyotropic CLCs. However, the phase separation was induced upon rising the temperature, resulting in the expulsion of MAA from the CLC helical molecular assemblage (Figure 5C, image *c*). This may be ascribed to the increase in the hydrophobicity of the EC derivatives arising from the complete etherification or increase in entropy because of the enhancement of the mobility of EC derivatives by their liquid crystallinity [34]. Therefore, the reflection color of the lyotropic CLCs was unchanged because of the preservation of CLC helical pitch length by the expulsion of MAA from the CLC helical molecular assemblage.

Furthermore, the decrease in the transmittance upon heating can be attributed to the light scattering from the aggregated scatterers of MAA with a size of 40–100 nm, as confirmed from the USAXS-SAXS intensity profiles.

## 3. Materials and Methods

### 3.1. Materials

EC was purchased from the Tokyo Chemical Industry (Tokyo, Japan). The viscosity of 5 wt% solutions of pristine ECs in the mixed solvent of toluene and ethanol (volume ratio 8:2) is 9–11 mPa·s according to the datasheet of the manufacturer. The number average molecular weight (*M*_n_) and weight average molecular weight (*M*_w_) of pristine EC were determined to be 2.58 × 10^5^ and 7.62 × 10^4^ by size-exclusion chromatography measurements in our previous work [27]. The molar amount of chemically combined ethylene oxide per anhydroglucose unit, that is, the molecular substitution (*MS*), was found to be 2.50 by the ^1^H-NMR spectrum measurement of pristine EC in CDCl_3_. Therefore, the average molecular weight per anhydroglucose monomer unit could be calculated to be 232. Before usage for the Williamson ether synthesis mentioned in the proceeding section, pristine EC was thoroughly dried under vacuum at room temperature for over 24 h.

Dehydrated *N*,*N*-dimethylacetoamide (DMAc) as solvent for etherification of EC is obtained from the Fujifilm Wako Pure Chemical Co., Inc. (Tokyo, Japan). 1-Bromopropane (PrBr), 1-bromobutane (BuBr), 1-bromopentane (PeBr), and 1-bromohexane (HeBr) used for the synthesis of etherified EC derivatives, MAA used as a solvent of lyotropic CLCs and 2-hydroxy-2-methylpropiophenone (HMPP) used as a photoinitiator were purchased from the Tokyo Chemical Industry (Tokyo, Japan). Sodium hydroxide (NaOH) and potassium iodide (KI), used as the catalysts of the Williamson ether synthesis, were acquired from the Fujifilm Wako Pure Chemical Co., Inc. (Tokyo, Japan). All reagents are except EC were used as received.

### 3.2. Synthesis and Characterization of the Completely Etherified EC Derivatives with Alkoxy Side Chains

The completely etherified EC derivatives were prepared by the Williamson ether synthesis as reported in our previous study [27]. Here the synthesis and characterization of propyl-etherified EC derivatives are described as an example (Supplementary Materials, Table S1, Sample Code: EC-Pr).

In a 100 mL round-bottomed flask, 3.00 g of EC was completely dissolved in 24.0 mL of dehydrated DMAc. After that, 4.01 mL of PrBr was added to the solution (5.00 eq. to hydroxy groups of EC). After stirring for 30 min at 65 °C, 2.40 g of powdered NaOH (0.10 g/mL for reaction solvents) and 0.27 g of KI (5.00 mol% to PrBr) were added. This reaction mixture was continuously stirred at 65 °C for 48 h. Subsequently, the reaction mixture was then purified via two rounds of centrifugation at 1.0 × 10^4^ rpm for 5 min to remove any sediment such as sodium bromide. Then, the supernatant was dialyzed against an equivolume mixture of methanol and water for 3 h, and the dialysis was prolonged for additional 48 h in an equivolume mixture of tetrahydrofuran and methanol by using a Visking dialysis tube with pore size of ~5 nm and a molecular weight cutoff between 1.2 × 10^4^ and 1.4 × 10^4^. The product was obtained through evaporation at 35 °C in vacuum for ~30 min and was finally dried for a few days to obtain purified EC-Pe.

The characterization of the EC derivatives was conducted by FT-IR spectroscopy equipped with an attenuated total reflection unit (FT-IR4700 and ATR PRO ONE, JASCO, Tokyo, Japan) and ^1^H-NMR spectroscopy (JNM-ECZ400S, JEOL, Tokyo, Japan) in line with our previous report [27].

### 3.3. Fabrication Procedure of Lyotropic CLC Cells

The EC derivatives were completely dissolved in MAA using a planetary centrifugal mixer (AR-100, THINKY, Tokyo, Japan). The lyotropic CLC mixture was sandwiched between two glass substrates. The cell gap was adjusted using polytetrafluoroethylene film spacers with a thickness of 200 µm. The edge of each cell was sealed with epoxy resin to prevent the evaporation of MAA upon the heating process.

### 3.4. Optical Measurement of Lyotropic CLC Cells

The transmission spectrum of visible light in the lyotropic CLC cells was measured on a compact charge-coupled (CCD) spectrometer (USB2000+, Ocean Optics, Orlando, FL, USA) equipped with an optical fiber. The CLC cell was illuminated with white light from a tungsten halogen light source (HL2000, Ocean Optics, Orlando, FL, USA) from the surface normal. The collinearly transmitted light from the CLC cell was focused through two pieces of achromatic doublet lenses, and it was collected into the entrance of an optical fiber connected with the CCD spectrometer. The temperature of the CLC cell was precisely controlled using a hot-stage system for the optical microscope (HS82 and HS1, Mettler Toledo, Columbus, OH, USA). Polarized optical microscope images were taken with a CCD camera (EO-5012, Edmund, Barrington, NJ, USA) equipped on the microscope (IX71, Olympus, Tokyo, Japan).

### 3.5. Preparation of UV-Cured Films from Lyotropic CLCs

To the lyotropic CLC mixtures of EC derivatives with MAA, a photoinitiator of HMPP was added at ~1.0 wt%. Subsequently, this CLC mixture was sandwiched between a pair of glass substrates. The cell gap was adjusted using polytetrafluoroethylene film spacers with a thickness of 500 µm. The cured films were prepared by irradiation of UV light at 365 nm from a compact light source equipped with light-emitting diodes (CL-H1-365-9-1, Asahi Spectra, Tokyo, Japan). The light intensity was set to be 80 mW cm^−2^ by using a photodiode sensor (PD300-UV, Ophir, Jerusalem, Israel). The temperature of the CLC cell was precisely controlled to be 30 °C, 70 °C, or 110 °C by using a temperature-controllable hot-stage system for the optical microscope (HS82 and HS1, Mettler Toledo, Columbus, OH, USA).

### 3.6. USAXS and SAXS Measurements of UV-Cured Films

USAXS and SAXS measurements were carried out at the BL19B2 beamline at the SPring-8 (Hyogo, Japan) using a photon counting detector (PILATUS 2M, Dectris, Baden, Switzerland) at a camera length of ~41 m or ~1 m. The X-ray was irradiated from the surface normal of CLC film at an energy of 18 keV for 60 s. The USAXS and SAXS intensities were corrected by transmission and subtraction of background scattering from air blank. The absolute intensity was calculated based on that of glassy carbon. In addition, the SAXS measurements were also conducted using a laboratory SAXS equipment (SAXSpace, Anton Paar, Graz, Austria) to connect the intensity profiles measured at the SPring-8.

## 4. Conclusions

In this study, we prepared the completely etherified EC derivatives possessing propyl, butyl, pentyl, or hexyl ether groups through the Williamson ether synthesis in order to investigate the dependence of the alkoxy side chain length on the light reflection properties of lyotropic CLCs with MAA. Butyl- or pentyl-etherified EC derivatives showed the lyotropic CLC phase with visible Bragg reflection when dissolved in MAA. Furthermore, it was found that the lyotropic CLCs of completely pentyl-etherified EC derivatives and MAA exhibited the most stable reflection color upon heating, regardless of the difference in their reflection wavelength. The USAXS and SAXS measurements of the polymerized films of lyotropic CLCs of etherified EC derivatives and MAA revealed that spherical scatterers are formed upon heating, indicating the phase separation between EC derivatives and MAA at the mesoscopic scale. Therefore, it is plausible that MAA molecules are excluded from lyotropic CLC structure so as to maintain the helical pitch length, corresponding to the reflection peak wavelength, by changing the temperature. This report provides a promising guideline to control the temperature dependence of the reflection peak wavelength of lyotropic CLCs, which is important from both scientific and technological aspects in the fabrication of environmentally and human-friendly photonic devices from cellulose derivatives.

## Figures and Tables

**Table 1:** Sample definition and preparation condition of lyotropic CLCs and their light reflection properties.

Sample	EC Derivatives	Polymer Conc. (wt%)	Reflection Wavelength at 30 °C (nm)	*λ*_shift_/10 °C (nm) *^a^*
1	EC-Bu	61.5	413	−3.0
2	EC-Bu	60.5	541	11
3	EC-Bu	59.2	640	15
4	EC-Pe	69.4	436	−1.4
5	EC-Pe	68.3	529	1.1
6	EC-Pe	67.4	632	5.9

*^a^* Average of reflection peak wavelength shift upon elevating the temperature range of 10 °C.

## Data Availability

Data are contained within this article.

## Supplementary Materials

The following supporting information can be downloaded at: https://www.mdpi.com/article/10.3390/molecules30132839/s1, Figure S1: Characterization of EC derivatives. Comparison of FT-IR spectra between pristine EC and etherified EC derivatives. ^1^H-NMR spectra of EC derivatives in CDCl_3_; Figure S2: Changes in transmission spectra of lyotropic CLCs upon the heating process from 30 °C to 110 °C. The measurements were conducted at 10 °C intervals. In the figures, the spectra at 30, 50, 70, 90, 100, and 110 °C are shown for reader’s clarity; Figure S3: USAXS-SAXS intensity profiles of UV-cured CLC films exhibiting green and red refection colors from the lyotropic CLCs of EC derivatives with MAA; Table S1. Synthesis conditions of etherified EC derivatives; Figure S4: Polarized optical microphotographs of lyotropic CLCs of EC-Bu or EC-Pe with MAA exhibiting blue refection colors under cross-Nicols.

## Abbreviations

The following abbreviations are used in this manuscript:

CLC	Cholesteric liquid crystal
EC	Ethyl cellulose
MAA	Methacrylic acid
FT	Fourier transform
IR	Infrared
NMR	Nuclear magnetic resonance
UV	Ultraviolet
USAXS	Ultra-small-angle X-ray scattering
SAXS	Small-angle X-ray scattering
POM	Polarized optical microscope
