# Supplementary material for: Insight into the Mechanism for the Emergence of Thermally Stable Reflection Colors from Cholesteric Liquid Crystals of Etherified Ethyl Cellulose Derivatives and Methacrylic Acid
Source: Molecules. 2025 Jul 2;30(13):2839. doi: 10.3390/molecules30132839 (PMC12250732; doi:10.3390/molecules30132839)
Supplement: Supplementary file 1 [file molecules-30-02839-s001.zip › molecules-3701596-supplementary.pdf]

Supplementary Materials

Insight into the Mechanism for the Emergence of Thermally Stable Reflection Color from Cholesteric Liquid Crystals of Etherified Ethyl Cellulose Derivatives and Methacrylic Acid

Wakako Kishi, Naoto Iwata* and Seiichi Furumi*

Department of Chemistry, Graduate School of Science, Tokyo University of Science,

1-3 Kagurazaka, Shinjuku, Tokyo 162-8601, Japan; 1325539@ed.tus.ac.jp

*Correspondence: n-iwata@rs.tus.ac.jp (N.I.); furumi@rs.tus.ac.jp (S.F.);
Tel.: +81-3-3260-4271 (N.I. & S.F.)

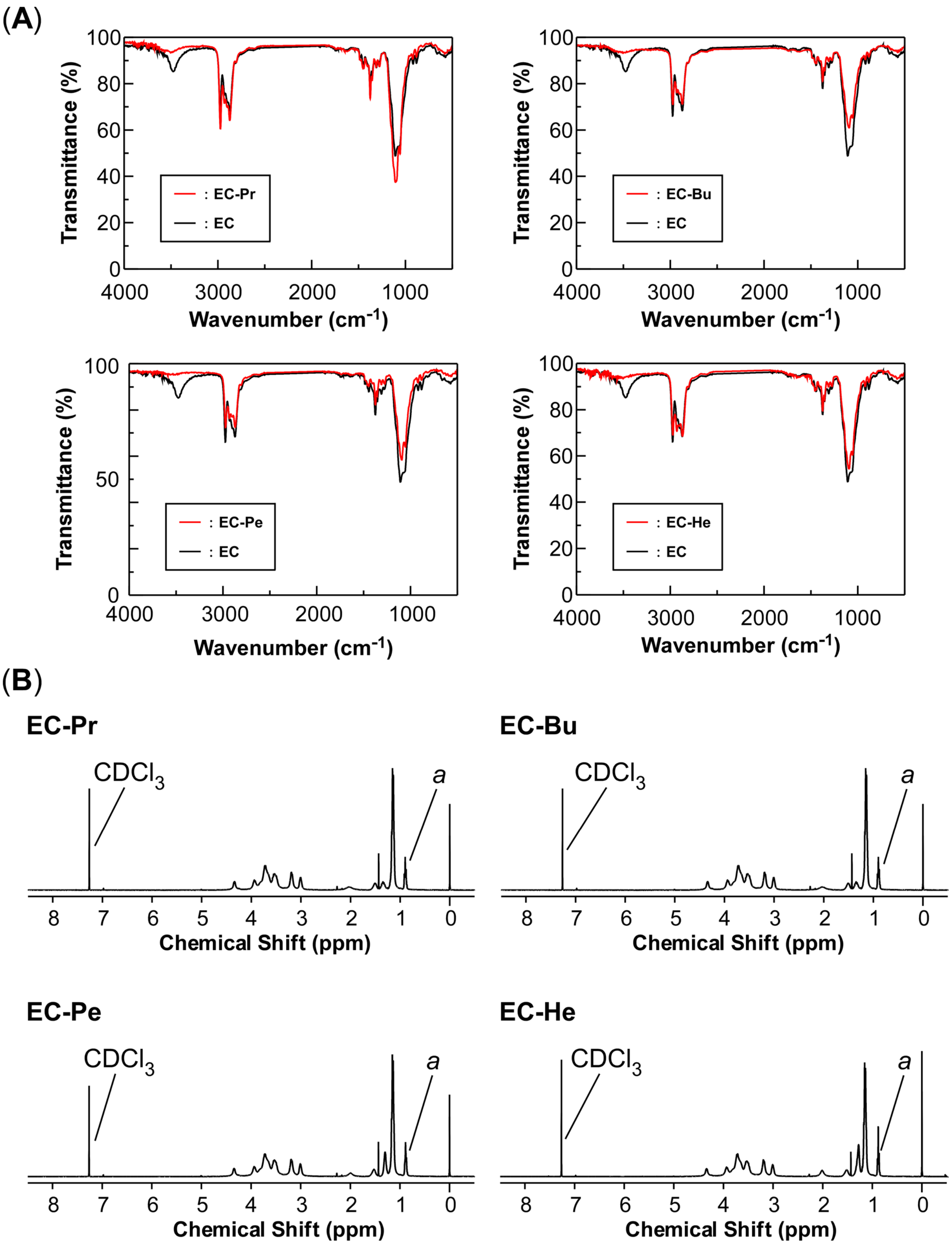

Figure S1. Characterization of EC derivatives. (**A**) Comparison of FT-IR spectra between pristine EC and etherified EC derivatives. (**B**) ^{1}H-NMR spectra of EC derivatives in $CDCl_3$.

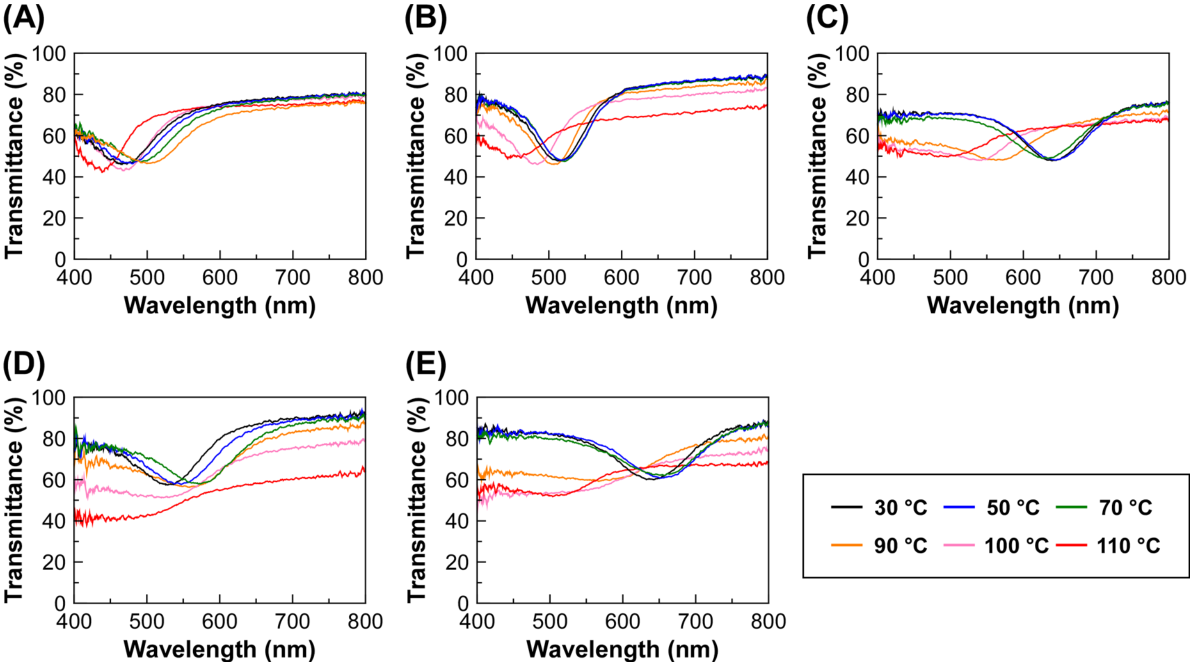

Figure S2. Changes in transmission spectra of lyotropic CLCs upon heating process from 30 °C to 110 °C. The measurements were conducted at 10 °C intervals. In the figures, the spectra at 30, 50, 70, 90, 100, and 110 °C are shown for reader's clarity. (**A**) **Sample 1**; (**B**) **Sample 2**; (**C**) **Sample 3**; (**D**) **Sample 5**; (**E**) **Sample 6**.

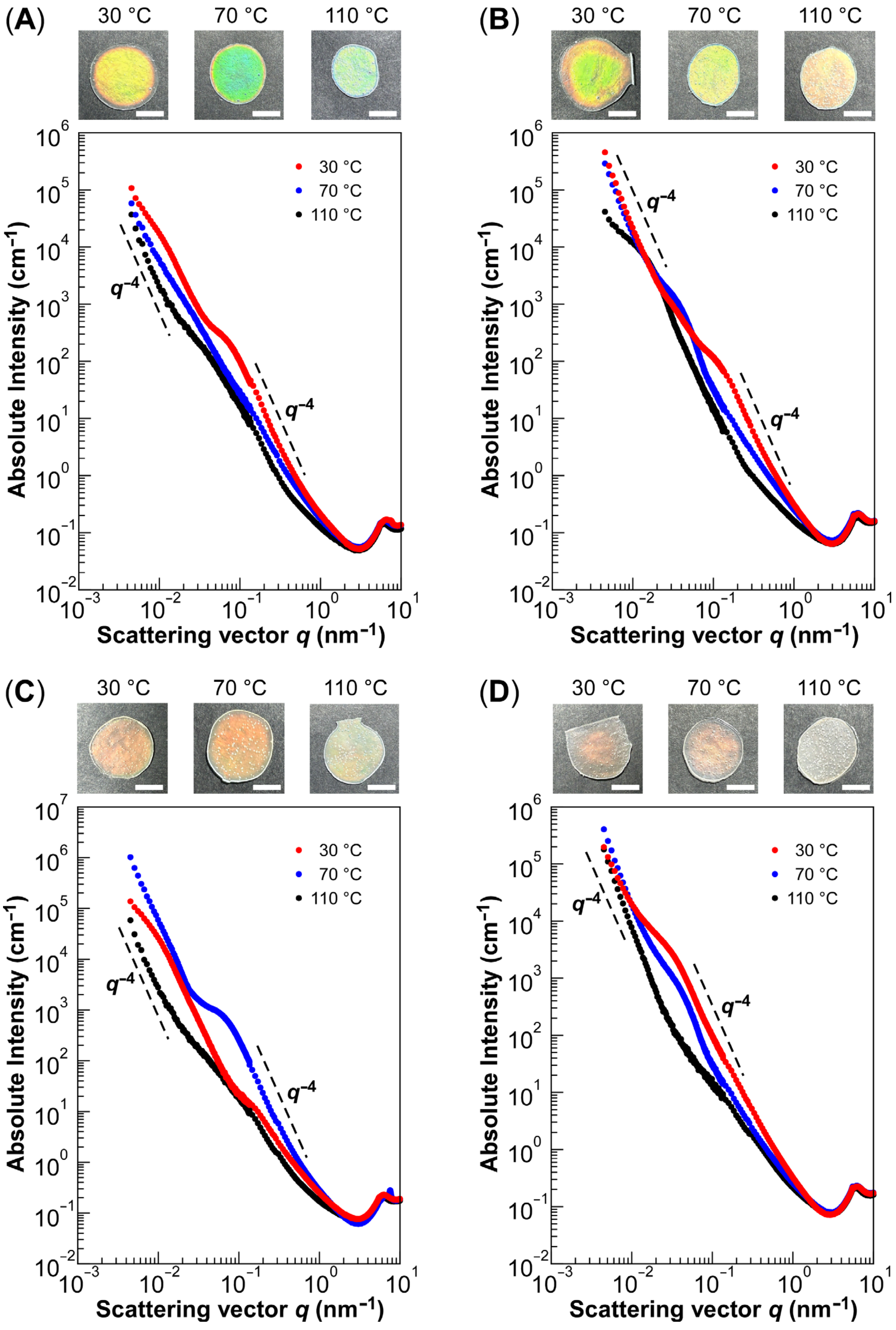

Figure S3. USAXS-SAXS intensity profiles of UV-cured CLC films exhibiting green and red refection colors from the lyotropic CLCs of EC derivatives with MAA. (**A**) UV-cured CLC films from **Sample 2**; (**B**) UV-cured CLC films from **Sample 5**; (**C**) UV-cured CLC films from **Sample 3**; (**D**) UV-cured CLC films from **Sample 6**. The insets show the reflection images of UV-cured CLC films, and the white scale bars denote 5 mm.

Table S1. Synthesis conditions of etherified EC derivatives.

Sample Code	Alkyl Bromide	NaOH Concentration (g/mL)
EC-Pr	PrBr	0.10
EC-Bu	BuBr	0.10
EC-Pe	PeBr	0.10
EC-He	HeBr	0.30

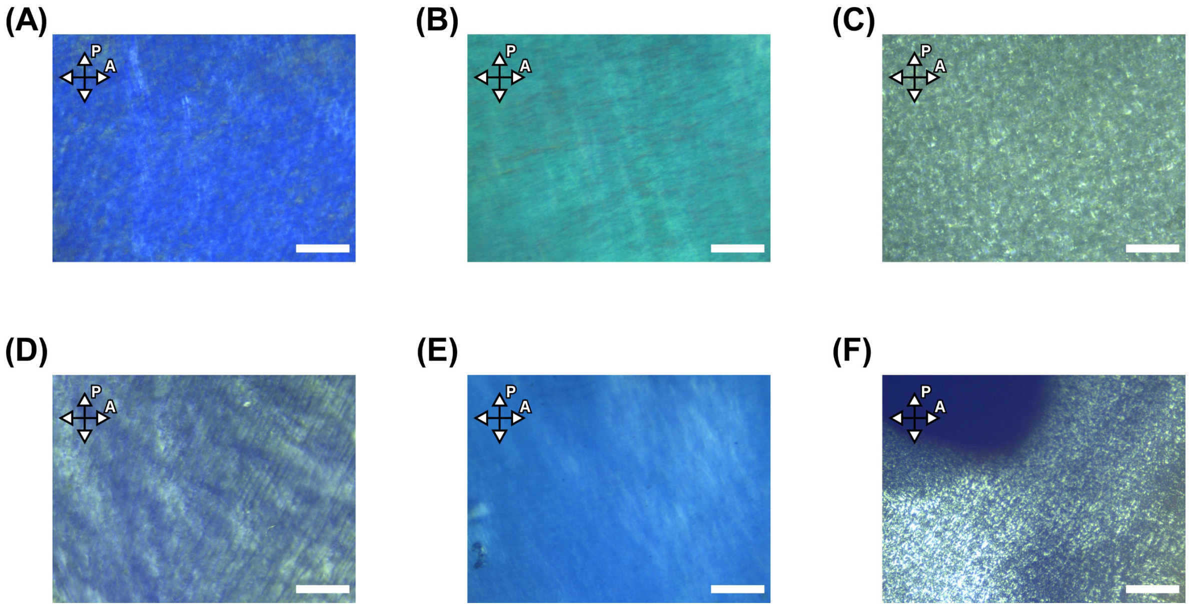

Figure S4. Polarized optical microphotographs of lyotropic CLCs of EC-Bu (**A**–**C**) or EC-Pe (**D**–**F**) with MAA exhibiting blue refection color under cross-Nicols. (**A**) EC-Bu with MAA at 30 °C; (**B**) EC-Bu with MAA at 70 °C; (**C**) EC-Bu with MAA at 110 °C; (**D**) EC-Pe with MAA at 30 °C; (**E**) EC-Pe with MAA at 70 °C; (**F**) EC-Pe with MAA at 110 °C. The white scale bars represent 500 μm.
